# A 54 Mb 11qter duplication and 0.9 Mb 1q44 deletion in a child with laryngomalacia and agenesis of corpus callosum

**DOI:** 10.1186/1755-8166-4-19

**Published:** 2011-09-21

**Authors:** Meena Lall, Seema Thakur, Ratna Puri, Ishwar Verma, Mithali Mukerji, Pankaj Jha

**Affiliations:** 1 Center of Medical Genetics, Sir Gangaram Hospital, Rajender Nagar, New Delhi 110024, India; 2 Genomics and Molecular Medicine, Institute of Genomics & Integrative Biology, Mall Road, New Delhi, India

**Keywords:** monosomy1q44, partial trisomy 11q, corpus callosum agenesis, laryngomalacia

## Abstract

**Background:**

Partial Trisomy 11q syndrome (or Duplication 11q) has defined clinical features and is documented as a rare syndrome by National Organization of Rare Disorders (NORD). Deletion 1q44 (or Monosomy 1q44) is a well-defined syndrome, but there is controversy about the genes lying in 1q44 region, responsible for agenesis of the corpus callosum. We report a female child with the rare Partial Trisomy 11q syndrome and Deletion 1q44 syndrome. The genomic imbalance in the proband was used for molecular characterization of the critical genes in 1q44 region for agenesis of corpus callosum. Some genes in 11q14q25 may be responsible for laryngomalacia.

**Results:**

We report a female child with dysmorphic features, microcephaly, growth retardation, seizures, acyanotic heart disease, and hand and foot deformities. She had agenesis of corpus callosum, laryngomalacia, anterior ectopic anus, esophageal reflux and respiratory distress. Chromosome analysis revealed a derivative chromosome 1. Her karyotype was 46,XX,der(1)t(1;11)(q44;q14)pat. The mother had a normal karyotype and the karyotype of the father was 46,XY,t(1;11)(q44;q14). SNP array analysis showed that the proband had a 54 Mb duplication of 11q14q25 and a 0.9 Mb deletion of the submicroscopic subtelomeric 1q44 region. Fluorescence Insitu Hybridisation confirmed the duplication of 11qter and deletion of 1qter.

**Conclusion:**

Laryngomalacia or obstruction of the upper airway is the outcome of increased dosage of some genes due to Partial Trisomy 11q Syndrome. In association with other phenotypic features, agenesis of corpus callosum appears to be a landmark phenotype for Deletion 1q44 syndrome, the critical genes lying proximal to *SMYD3 *in 1q44 region.

## Background

Partial Trisomy 11q syndrome is a rare disorder with defined clinical features and has been documented in the list of rare syndromes by National Organization of Rare Disorders (NORD), USA http://www.raredisease.org[1]. These clinical features consist of distinct pattern of facial features, mental retardation, pre- and postnatal growth retardation, hypotonia, congenital heart defects and limb malformations [1-3]. There is one report [4], which states that the malformation of the upper airway is associated with trisomy 11q.

Deletion 1q44 syndrome is a well-recognized syndrome archived in the National Medical Library http://www.nlm.nih.gov/archive/20061212/mesh/jablonski/mesh/jablonski/syndrome_db.html[5]. The clinical features of 1qter syndrome include short stature, developmental delay, mental retardation, microcephaly, seizures, an abnormal corpus callosum ranging from partial to complete agenesis, and abnormal ear shape [5-7]. Genomic imbalances cause a clinical phenotype in a patient depending on the size and content of genes in the aberration. There is controversy about the genes in 1q44 responsible for agenesis of the corpus callosum [8-14].

We present a case report of a female child with 54 Mb of 11qter duplication and 0.9 Mb of 1q44 deletion. The clinical findings are compared with previously reported patients. This genomic imbalance in the proband was used to delineate the critical genes that possibly contribute to laryngomalacia and agenesis of the corpus callosum in proband.

## Clinical Presentation

### A Case Report

A female child was born as the product of the second pregnancy to a healthy non-consanguineous couple at 37 weeks of gestation. The mother was 28 years old and the father was 31 years old. Her delivery was by Caesarean section in view of respiratory distress. Her birth weight was 2.38 kg, length 47 cms and head circumference 31 cms. She developed septicemia. The total leukocyte counts were raised (47,000/cumm), the platelets were lowered (54,000/cumm) and the blood culture showed staph aureus. Omnatax and Amikacin were started. The baby had jaundice. She had stridor, which disappeared, in prone position. She was given intravenous fluids. She improved gradually but developed abdominal distension and vomiting on the fourteenth day and was lethargic. She was continued to be on antibiotics. She slowly recovered but had difficulty in swallowing. Therefore, she was given feeds through Ryle's tube.

**Figure 1:**
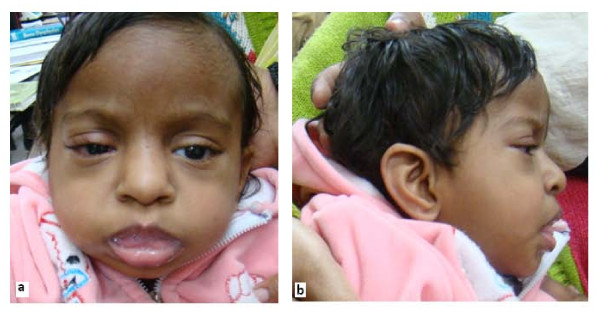
**Patient facial features**. Round face with heavy cheeks and prominent forehead, upward slanting eyes, palpebral fissures, epicanthic folds, broad bulbous nose, large dysplastic ears, long and smooth philtrum, down turned corners of the mouth, thin tented upper lips, short neck.

**Figure 2:**
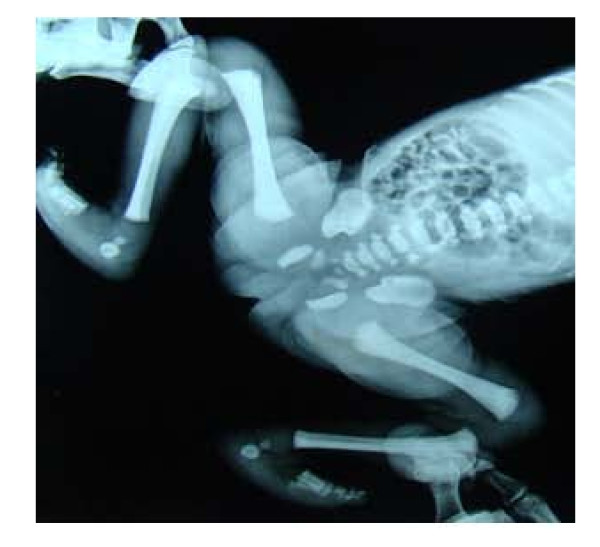
**Rocker bottom feet visible at birth -visualized in the X-ray, during the retrospective study**.

**Figure 3:**
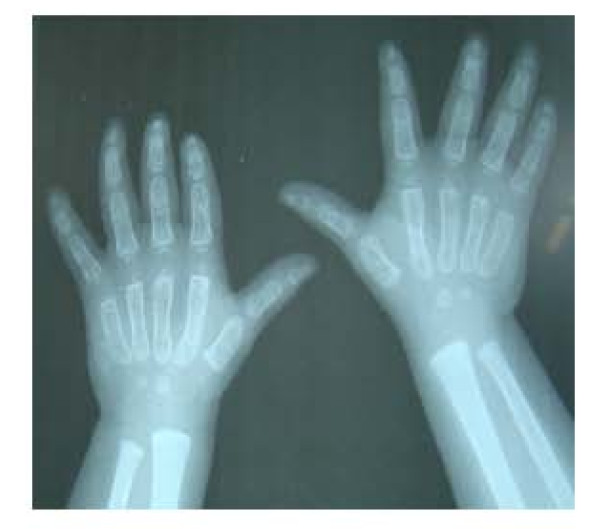
**X-ray hands show evidence of osteopenia and uneven fingers**.

She had dysmorphic features (Figure 1a and 1b) with microcephaly and hypotonia. Her face was round with heavy cheeks and prominent forehead, upward slanting eyes, palpebral fissures, epicanthic folds, broad bulbous nose, large and low set ears, long and smooth philtrum, and thin tented upper lip. She had rocker bottom feet (Figure 2), uneven fingers and X-ray showed evidence of osteopenia.(Figure 3).

**Figure 4:**
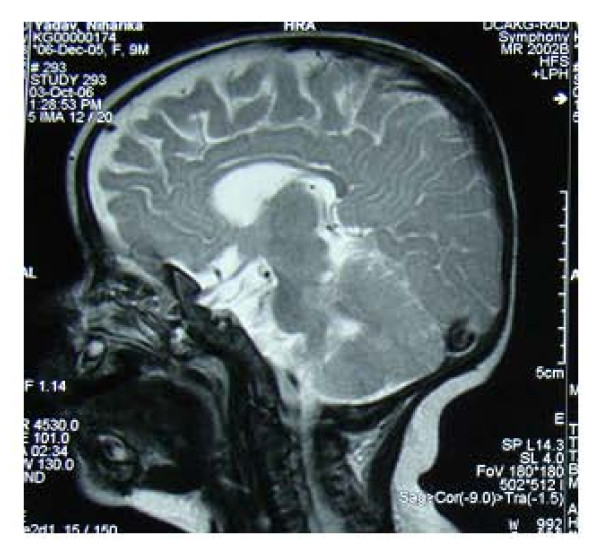
**Partial agenesis of corpus callosum**.

She had a cat-like cry, laryngomalacia, difficulty in swallowing, esophageal reflux and anterior ectopic anus, for which she was operated. She also had a murmur for which ECHO was done. This revealed atrial septal defect and patent ductus arteriosus of small size. The brain magnetic resonance imaging showed that the corpus callosum was diffusely thin for age with altered signals and suggestive of partial agenesis (Figure 4).

She was reviewed again at two years of age. She had history of multiple admissions with fever and respiratory distress and episodes of seizures at 10 months. The history of difficulty in swallowing during the neonatal period had gradually improved, but there was history of choking when solids were introduced after 6 mths. Her milestones were delayed: head holding at 1 year 10 mths. She rolled from supine to prone at 1 year and could never hold an object in her hand. She gave a social smile at 2 years.

**Figure 5:**
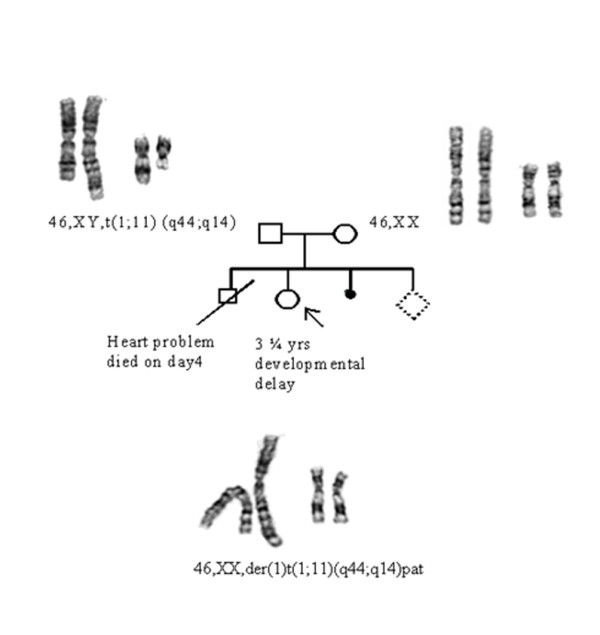
**Pedigree: 1st child-abnormal, no karyotyping done, child died on day 4**. 2^nd ^child- proband 3 1/2 years, developmental delay. 3^rd ^child -spontaneous abortion. 4^th ^fetus has balanced translocation. Father is a carrier of balanced translocation and mother has a normal karyotype.

The first pregnancy of the mother was a live born male, who had a heart disease and died after four days after birth. No karyotyping or any other evaluation of this child was available. The third pregnancy was terminated in a spontaneous abortion. The mother is now 16 weeks pregnant. Her pregnancy is being monitored. There is no other family history of congenital malformations or mental handicap. A pedigree of the family is shown in Figure 5.

## Methods

Peripheral blood lymphocyte cultures of the proband, the mother and the father were set up by standard technique [15] for karyotyping with high resolution GTG-banding. Chromosome analyses were done in twenty metaphases for each sample with a resolution of 500-550 bands. The karyotypes were described in accordance with the international system for human cytogenetic nomenclature (ISCN, 2009) [16].

Subtelomeric FISH analyses for chromosomes 1 and 11 were done [17] using the corresponding commercially available DNA probes (Vysis, Abott Laboratories) on the cultured lymphocytes of the proband.

250 ng of DNA was isolated from peripheral blood of the proband using the QIAGEN QIAamp Midi kit (Qiagen, Valencia, CA), according to the manufacturer's instructions. DNA concentration and the quality were checked using a NanoDrop ND-1000 spectrophotometer, before processing for SNP genotyping. The extracted DNA was processed with the Illumina human 610-quad v1.0 genotyping bead chip, according to the manufacturer's instruction for the Infinium HD Assay protocol. This is SNP based technology [18] with integrated hidden Markov model designed for high-resolution copy number variation detection in the whole-genome SNP genotyping data [19]. The Human 610-quad bead chip was imaged on the Illumina Bead Array Reader and the data was processed with both Illumina Genome Studio v2009.1 and KaryoStudio v.1.0.3 software modules. The SNP data was analyzed for copy number variations (losses or gains) and the size of the aberration was recorded. The gene content was examined using http://genome.ucsc.edu/cgi-bin/hgTracks or UCSC genome browser website [20]

## Results

GTG-banded chromosome analysis at 500-550 band resolution revealed a derivative chromosome 1 in the proband. Her karyotype was 46,XX,der(1)t(1;11)(q44;q14)pat. The mother had a normal karyotype and the karyotype of the father was 46,XY,t(1;11)(q44;q14).

**Figure 6:**
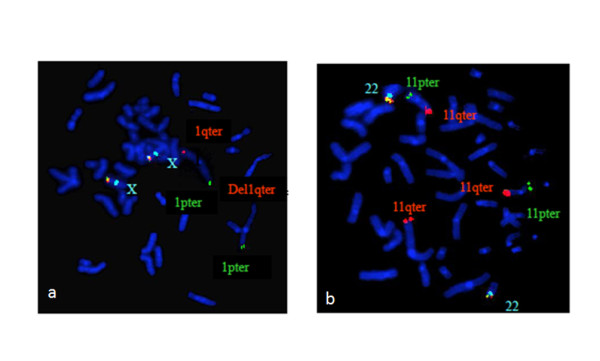
**Subtelomeric DNA probe for chromosomes**. a. Subtelomeric DNA probe for Chromosome 1p (spectrum green) showed two green signals and subtelomeric DNA probe for Chromosome 1q (spectrum orange) showed one orange signal denoting that subtelomeric region of 1q is deleted causing monosomy 1qter. Multiple colored probe of the X chromosome served as control for this FISH test. b. Subtelomeric DNA probe for Chromosome 11p (spectrum green) showed two green signals and subtelomeric DNA probe for Chromosome 11q (spectrum orange) showed three orange signal denoting that there is trisomy of the distal 11qter region. Multiple colored probe of the chromosome 22 served as control for this FISH test.

Subtelomeric FISH analyses of chromosome 1 (Figure 6a) showed two signals for 1pter and only one signal for 1qter denoting that there was a deletion of 1q44. Subtelomeric FISH analyses of chromosome 11 (Figure 6b) showed two signals for 11pter and three signals for 11qter denoting that there was a trisomy of 11qter. The derivative chromosome 1 carried the deletion of 1qter region and duplication of 11qter.

**Figure 7:**
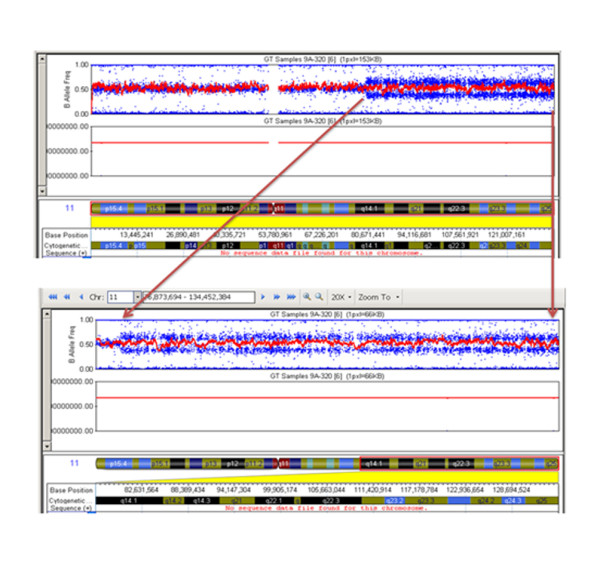
**Illumina Infinium 610 Quad SNP array analysis showed ~ 54 Mb duplication in region11q14.1-11q25 (chr11: 79,512,964 - 134,452,384) on chromosome 11**.

**Figure 8:**
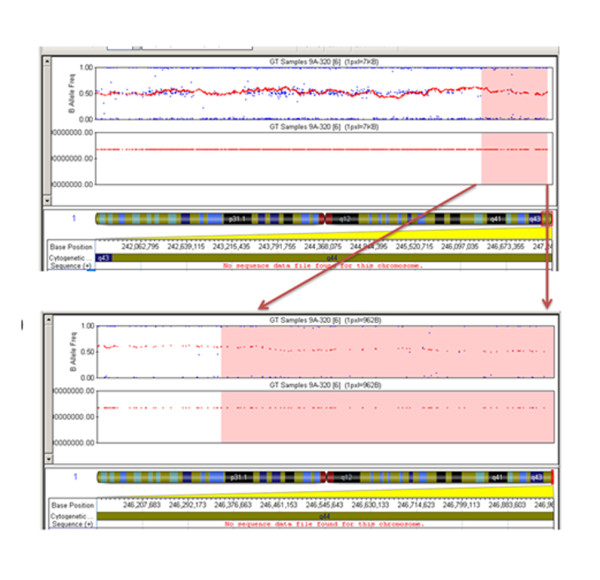
**Illumina Infinium 610 Quad SNP array analysis showed ~ 0.9 Mb deletion in region 1q44 (chr1: 246,352,064-247,185,943) on chromosome no.1**.

SNP array analysis showed a 54 Mb duplication in the region 11q14q25 (chr11: 79,512,964 - 134,452,384) on chromosome 11(Figure 7) and a 0.9 Mb deletion in region 1q44 (chr1: 246,352,064-247,185,943) on chromosome no.1 (Figure 8).

## Discussion

The proband had inherited the derivative chromosome 1 as an unbalanced translocation from the father who was a balanced translocation carrier. The proposita was trisomic for 54 Mb of 11q14q25 region and also monosomic for 0.9 Mb submicroscopic subtelomeric 1q44 region.

Therefore she had two syndromes: a rare Partial Trisomy 11q syndrome and Monosomy1q44 syndrome. The phenotypes are tabulated and compared with similar published data (Table 1)

**Table 1:** The phenotype of the proband was compared with the clinical features for Monosomy 1qter syndrome and partial trisomy 11q syndrome (as delineated by previous reports)

Monosomy 1qter syndrome **Previous reports **[2,4-7]	Proband	Partial Trisomy 11q **Previous reports **[1,3]	Proband
**General**		General	

Mental retardation	+	Mental retardation	+

Growth retardation	+	Pre and post natal growth retardation	+

Microcephaly	+	Microcephaly	+

Hypotonia	+	Hypotonia	+

Seizures	+	Short stature	_

**Face**		Delayed milestones	+

Sparse fine hair	+		

Prominent forehead/metopic ridge	+		

Upward slanting palpebral fissures	+	Slanted palpebral fissures	+

Epicanthic folds	+	Epicanthic folds	+

Strabismus	_		

Flat nasal bridge	+		

Short, broad nose	+	Short nose	+

Smooth, long philtrum	+	Long philtrum	+

Thin vermilion	+	Retracted lower lip	_

Downturned corners of the mouth	+	High arched palate	_

Micrognathia	+	micrognathia	+

Cleft palate	_	Low set ears	+

Abnormal ears	+	Dysplastic ears	+

**Other**			

Short neck	+	Short neck	+

Cardiac anomaly	+	Heart defect	+

Abnormal hands	+	micropenis	

Abnormal feet	+	Dislocation of the hips	_

Gastro-oesophageal reflux	+	Neonatal feeding problem	+

**Corpus callosum agenesis/hypoplasia**	**+**	**Recurrent upper airway infections**	**+**

Trisomy 11q was first referred as a distinct clinical entity in 1977 and was referred to as duplication 11 (q21-23) syndrome [2]. This trisomy mostly occurs along with any other monosomy due to an unbalanced translocation, as it has occurred in the proband of the present study. Most patients are reported to have partial trisomy 11q due to the translocation t(11;22) [21]. Dominique et al (1997) [3] compared the clinical features of patients with pure trisomy 11qter and those with additional chromosomal anomalies and revealed a set of common clinical features in this syndrome. These clinical features have also been documented by NORD [1]: mental retardation, pre- and postnatal growth retardation, hypotonia, distinct pattern of facial features, congenital heart defects and limb malformations. The clinical findings of the proband were compared and found to be similar to this published data (Table 1).

Hui-quan Zhao et al, 2003[4] reported the upper airway obstruction secondary to a malformed epiglottis and suggested that the critical region for this malformation is 11q21q23. Laryngeal Atresia is a rare congenital malformation [22]. This anomaly is one of the etiologic factors causing congenital laryngeal high airway obstruction syndrome. The proband of the present study was trisomic for the region 11q14q25. Besides all the above-mentioned clinical criteria, she had a cat like cry, difficulty in swallowing, laryngomalacia and also recurrent episodes of respiratory distress, since birth, showing that the upper airway malformation was present. Therefore laryngomalacia or obstruction of upper air way may be considered as one of the clinical features in Partial Trisomy 11q syndrome. 0.54 Mb in the 11q14q25 region contains a large number of genes. It is not possible to pinpoint at any one particular gene responsible for laryngomalacia. However, one can conclude that increased gene dosage in this region may be responsible for this phenotype.

1qter microdeletions are often reported as part of a complex chromosome rearrangement and few de novo isolated 1qter microdeletions have been reported [23]. The recognizable phenotype for submicroscopic 1qter deletion is archived in NML NIH USA [5]. The proband had most of these features as shown in the table 1. Several abnormalities in this syndrome are related to the midline, such as agenesis or hypoplasia of the corpus callosum, cardiac anomalies, genital anomalies and gastro esophageal abnormalities [6,7]. The genes involved in normal midline development might be located in the deleted region of 1q44 [6,7].

**Figure 9:**
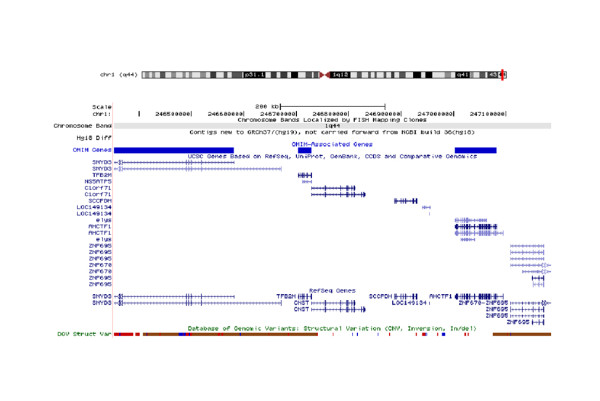
**Genes in the deleted 0.9 Mb deleted region in 1q44 (http://genome.ucsc.edu/cgi-bin/hgTracks website)**.

Numerous 1qter deletions have already been described associated with brain malformations along with other recognizable phenotypes. But there is a controversy about the genes in 1q44 region responsible for agenesis of the corpus callosum [Table 2]. Van Bever et al, 2006 [24] attempted the first molecular characterization of deletion 1q44. They reported a deletion of 4.5 Mb mapped within *RSG7 *in 1q44. Boland et al (2007) [9] described mapping studies of patients with unbalanced structural rearrangements of distal 1q44. They concluded that *AKT3 *haplo-insufficiency causes both postnatal microcephaly and agenesis of the corpus callosum. Joris et al (2008) [10] suggested that among 1q44 deleted genes, *AKT3 *is a strong candidate gene for vermis hypoplasia and corpus callosum agenesis. Orellana et al, 2009[11] also supported the role of *AKT3 *and *ZNF238 *in their study of 1.1 Mb deletion in 1q44 region. However, Van Bon et al, 2008[12], through detailed molecular analysis of the deletion sizes in their 13 patients, showed that a 360 kb genomic segment contained the candidate genes for the critical region for corpus callosum, but excluded the *AKT3 *haploinsufficiency. Poot et al, 2008 [25] also rejected the *AKT3 *and *ZNF238*, as both the genes were not deleted in their study of a 4.8 deletion in 1q44 and the phenotype-included agenesis of corpus callosum. Aktas et al (2010) [13] detected a 2.7 Mb deletion in 1q44 starting from 244444664 bp to 247110269 bp, the distal breakpoint was located in *ZNF672 *and the proximal breakpoint was located in *SMYD3*. They concluded that Corpus callosum development is dependent on the critical genes lying in the short segment of 300 kb between the *C1orf100 *and *C1orf121 *in 1q44. The present study reports a 1q44 deletion overlapping with the deletion reported by Aktas et al, 2010. The proband had a smaller deletion of 0.9 Mb in 1q44 starting from 246,352,064 bp to247, 185,943 bp, the distal breakpoint was located in *ZNF695 *and the proximal breakpoint was located in *SMYD3 *gene as viewed in the UCSC genome browser http://genome.ucsc.edu (Figure 9). *AKT3 *was not present in the 0.9 Mb deleted region at 1q44 but the proband had agenesis of the corpus callosum.

**Table 2:** Candidate genes for agenesis of corpus callosum

Publication	Deletion in 1qter	Proposed Candidate gene
Van Bever et al, 2005[8]	4.5 Mb in1q44	Mapped within *RSG7*

Boland et al, 2007[9]	3.5 Mb in1q44	AKT3, ZNF238

Joris et al, 2008[10]	6.9 Mb in1q44	*AKT3 *strong candidate gene

Van Bon et al, 2008[12]	0.36 Mb in1q44	*C1orf100, ADSs, C1orf101 *&*C1orf121*. *AKT3 *gene was excluded

Poot et al, 2008[25]	4.8 Mb in1q44	*AKT3, ZNF238 *was not deleted Therefore *AKT3 *was rejected

Orellana et al, 2009[11]	1.1 Mb in1q44	AKT3, ZNF238, C1orf100

Aktas et al, 2010[13]	2.9 Mb and 2.7 Mb in1q44	*C1orf100 *to *C1orf121* Genes proximal to *SMYD3* *AKT3 *was excluded

Caliebe et al, 2010[14]	0.44 Mb in1q44	*FAM36A, HNRPU, EFCAB2, KIF26B*

Osburn et al,2011[8]	Deletion in 1q42.13 to q44	DISC1

Lall M et al,2011 [present study]	0.9 Mb in1q44	Genes proximal to *SMYD3* *AKT3 *excluded

Calibe et al 2010[14] has showed four patients with speech delay, seizures and variable corpus callosum thickness sharing a 0.44 Mb deletion in 1q44, which contained *FAM36A, HNRPU, EFCAB2 and KIF26B *genes. It was hypothesized that *HNRPU *is involved in the regulation of embryonic brain development, so it represents a novel plausible candidate. Osburn et al, 2011[8] reported a de novo chromosome deletion at 1q42.13 to q44, which includes *DISC1*, in an individual with agenesis of corpus callosum. They also showed that the developmental expression of mouse DISC1 is highly expressed in the embryonic corpus callosum at a critical time for callosum formation. There is couple of other reports [26,27] of deletions in 1q42-q43 region associated with corpus callosum agenesis. Therefore if genes aligned all along 1q42-q44 are responsible for the development of the corpus callosum, there may be a contiguous gene effect. The genes contained within the deleted region may not always be responsible for the pathology but the altered expression levels of genes located in the vicinity may be the cause. Therefore position effects and possible interactions with other loci should also be considered.

## Conclusion

Laryngomalacia or obstruction of the upper airway is related to gene dosage effect due to Partial 11q Trisomy Syndrome. With all other well-documented phenotypic features, agenesis of corpus callosum is an important phenotype in Deletion 1q44 Syndrome, the critical genes lying in 1q44 proximal to *SMYD3. AKT3 *is excluded. These findings and review of recent reports suggest that position effect and possible interactions with other loci may be responsible for agenesis of corpus callosum.

## Consent

Written informed consent was obtained from the parent of the patient for publication of this case report and any accompanying images. A copy of the written consent is available for review by editor-in chief of this journal.

## List of abbreviations used

NORD: National Organization of Rare Disorders; FISH: fluorescence Insitu Hybridisation; ISCN: international system for human cytogenetic nomenclature

## Competing interests

The authors declare that they have no competing interests.

## Authors' contributions

LM wrote the manuscript, performed the cytogenetic analysis, FISH analysis and SNP array and their interpretation. TS, PR, VIC carried out clinical examination and evaluation. JP MM performed SNP array analysis and interpretation. All authors read and approved the final manuscript.
